# Improvement of out-of-hospital cardiac arrest survival rate after implementation of the 2010 resuscitation guidelines

**DOI:** 10.1371/journal.pone.0204169

**Published:** 2018-09-24

**Authors:** Robert Larribau, Hélène Deham, Marc Niquille, François Pierre Sarasin

**Affiliations:** Division of Emergency Medicine, Department of Community Medicine, Primary Care and Emergency Medicine, Geneva University Hospitals, Geneva, Switzerland; Medizinische Hochschule Hannover, GERMANY

## Abstract

**Objective:**

The implementation of cardiopulmonary resuscitation guidelines, updated every five years, appears to improve patient survival rates after Out-Of-Hospital Cardiac Arrest (OHCA). The aim of this study is: 1) to measure the level of improvement in the prognosis of OHCA patient survival rates for the years 2009 and 2010 and the following two years 2011 and 2012; and 2) correlate the improvement in prognosis with the updated 2010 Advanced Cardiovascular Life Support (ACLS) Guidelines.

**Method:**

We performed a retrospective observational study based on Geneva’s OHCA register that includes data from January 1, 2009 to December 31, 2012. We compared the evolution of prognostic factors that influenced survival at hospital discharge between the periods before and after the implementation of the 2010 guidelines. We then compared the survival rates between each period. Finally, we adjusted the effects on survival in the second period to prognostic factors not linked with the care provided by Emergency Medical Services (EMS) teams, using a multivariable logistic regression model. Changes in advanced resuscitation treatment provided by EMS personnel were also examined.

**Results:**

795 OHCA were resuscitated between 1^st^ January, 2009 and 31^st^ December, 2012. The prognosis of patient survival at the time of hospital discharge rose from 10.33% in 2009–2010 to 17.01% in 2011–2012 (p = 0.007). After making adjustments for the effect of improved survival rates on the second period with factors not related to care provided by EMS teams, the odds ratio (OR) remains comparable (OR = 1.87, 95% CI [1.08–3.22]). Measured changes in treatment provided by EMS personnel were minor.

**Conclusions:**

Survival rate for OHCA patients improved significantly in 2011–2012. This study suggests that it was probably the improvement in the quality of care provided during CPR and post-cardiac arrest care that have contributed to the increase in survival rates at the time of hospital discharge.

## Background

The overall prognosis after Out-Of-Hospital Cardiac Arrest (OHCA) in an Advanced Life Support (ALS) system remains poor, with a survival rate of around 10% following hospital discharge, no matter what the cause of OHCA [1, 2]. There is, however, a slow improvement in both shockable (VF / VT) OHCA and non-shockable OHCA, which has resulted in a doubling of survival rates over the last 30 years [3, 4].

How survival rates are affected by emergency care and treatment provided to OHCA patients is poorly understood. Immediate chest compressions and early defibrillation are the only emergency care procedures that have clearly demonstrated a positive effect on likely survival rates [5].

Globally, it was noted that the improved prognosis was linked to implementation of the international recommendations for cardiopulmonary resuscitation [4–12]. Cardio-Pulmonary Resuscitation (CPR) and emergency care have developed over many years and were revised regularly [13]. In 2000, the first "universal" guidelines for CPR proposed evidence based treatments ranked according to the level of scientific evidence. This rapidly modified the emergency care provided by EMS personnel [8, 9]. In the 2005 guidelines, a new compression-ventilation ratio of 30:2 was introduced for all victims [10]. In addition, the three-stacked shocks sequence was abandoned for a one-shock protocol, followed by the immediate resumption of effective chest compressions without immediate pulse check [14, 15].

**Fig 1:**
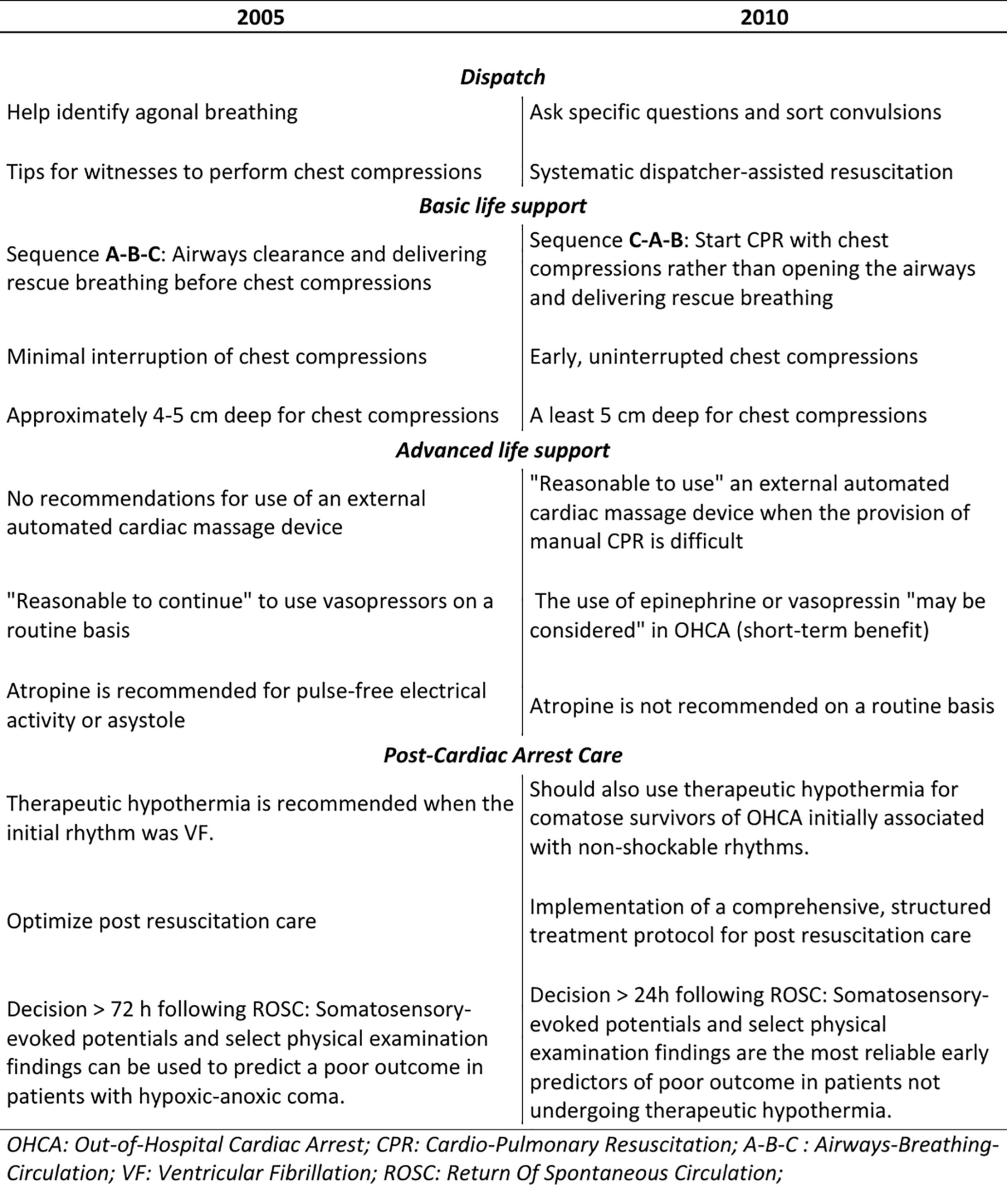
Major changes between 2005 and 2010 resuscitation guidelines.

At the end of 2010, new versions of Basic Life Support (BLS) and Advanced Cardiovascular Life Support (ACLS) Guidelines (“2010 ACLS Guidelines”) for CPR were published [11, 12]. The main changes between the 2005 and 2010 resuscitation guidelines are described in the Fig 1.

In 2010 Guidelines, chest compressions, which replace the pumping action of the heart, must be started as soon as there is the slightest suspicion of cardiac arrest and should be continuous and uninterrupted in order to avoid cerebral low flow [16]. In 2010 ACLS Guidelines, the introduction of a new additional phase of "immediate post-resuscitation care" to the "chain of survival" was intended to emphasize that advanced care continues well beyond the time when the patient's heart returns to spontaneous circulation [17]. Furthermore, it is believed that post-resuscitation care may also improve the patient’s neurological state [18].

Whilst the effects of implementing the 2005 Guidelines on OHCA patients have been relatively well documented, no studies appear to have been specifically designed to measure the impact of the revision of the 2010 ACLS Guidelines on the evolution of survival rates of OHCA patients [3, 4, 19–21]. However, there is a recent Japanese study measuring the impact of the 2010 Guidelines but which only examined the impact in an “Intermediate Life Support” system in Japan. This study showed an improvement of survival rates at one-month after hospital discharge, following the implementation of the 2010 Guidelines [22].

The main objective of this study was therefore to analyse the evolution of patient survival at the time of hospital discharge between period 1 (2009–2010), and period 2 (2011–2012) and to correlate this evolution with the changes incorporated in the 2010 ACLS Guidelines.

The secondary objectives were to measure the impact of the 2010 ACLS Guidelines on: 1) the number of patients who recover a Return Of Spontaneous Circulation (ROSC); and 2) the number of patients admitted to hospital whether they experienced ROSC or not.

## Materials and methods

### Design of the study

The first step of the study was to identify which prognostic factors influence OHCA patient survival rates at the time of ROSC on hospital admission and discharge. Four groups of prognostic factors were defined in accordance with the 2015 Utstein template [22], that is:

1. Prognostic factors associated with patients’ characteristics and the situation at the time the OHCA occurs;
2. prognostic factors related to emergency medical dispatch;
3. prognostic factors related to action taken by bystanders; and
4. prognostic factors related to emergency treatment provided by EMS personnel.

Once the factors that had a significant impact on survival rates had been identified, the second step was to measure any changes to those factors after implementation of the 2010 Guidelines [11].

For each of the periods studied, the third step was to measure the number of patients who experienced ROSC, who were admitted to hospital and those who had survived at the time of hospital discharge. In this way it was possible to compare survival rates for each period and establish the differences between them.

Finally, prognostic factors that significantly impacted survival, and which were not immediately modified by the implementation of the 2010 Guidelines (patient characteristics, situation at the time the OHCA occurs, emergency medical dispatch, response times), were included in a logistic regression adjustment model in order to show the influence of the “before and after” period on survival. The hypothesis being that, in the absence of any confounding effects related to these variables, the change in prognosis was due to changes in emergency treatment provided by EMS personnel following the publication of the 2010 Guidelines [11].

Of note: The new ERC guidelines for cardiopulmonary resuscitation, published in mid-October 2010, were implemented in our local emergency medical services, as early as January 2011 for the majority of cardiac arrest situations. Although most of the personnel from the seven ambulance services were trained in November and December 2010, some paramedics, who were not present during that period, were trained in January and February 2011; for this reason we include the four-month transition period.

### Geneva’s EMS

The Canton of Geneva, covering an area of 282.48 km2, is a predominantly urban Canton, with a population of 470'510 in 2012. At that time, 21.12% of Geneva residents were under the age of 20 years, and 16.24% over 64 years; 50.60% were women. In addition to the local population, according to 2012 statistics [23], there were about 130,000 daily commuters coming in to work in Geneva from both neighbouring cantons and France.

At the time of the study, a single emergency medical communication centre received all emergency calls for OHCA directly. Paramedics or nurses from the call centre handled all calls, assessed situations with a criteria-based dispatch system. Following assessment, they contacted both available advanced life support (ALS) response levels describe below closest to the location of the OHCA launched the emergency intervention.

The first ALS level consisted of a fully equipped ambulance with two paramedics who were trained to carry out the ALS autonomously following strictly defined protocols, with the exception of orotracheal intubation. The ambulance services maintain a dozen vehicles, stationed at ten different bases throughout the canton of Geneva.

The second ALS level, engaged simultaneously if an OHCA was identified during the call, consisted of a light vehicle with a paramedic and a doctor on board. This second ALS level carried an automated external cardiac massage device on board. There was one vehicle based in the centre of Geneva and this second ALS level intervened in more than 90% of OHCA in the canton. During the day, and if necessary, a medical helicopter could also be mobilised. Finally, a supervising physician was available to intervene should reinforcements be required or to replace of the second ALS level if it was already occupied.

To ensure that the guidelines are correctly implemented in our emergency medical system, strict quality controls are imposed through the use of protocols. Once a day, a senior physician reviews the resuscitation forms in the presence of the pre-hospital team having provided resuscitation in order to ensure that the resuscitation Guidelines have been strictly applied. When the patient was transported to hospital, he was always admitted to the emergency department of Geneva University Hospitals, except in a situation where a pre-hospital alarm for suspected heart infarct had been triggered. In this event, the patient was sent directly to the catheterization laboratory in the same hospital.

After 48 to 72 hours in the hospital, and if the patient remained comatose, his neurological status was evaluated according to a strict evaluation protocol. This protocol involved performing Magnetic Resonance Imaging of the brain, an electroencephalogram coupled with a selected physical examination, and somatosensory evoked potentials. Depending on the results of these examinations, a decision was taken (72 hours after admission) as to whether or not the treatment should be stopped. Stopping the treatment was a common scenario in cases of vegetative coma. The international guidelines for post-resuscitation hospital care were applied, with the exception of induced Therapeutic Hypothermia (TH between 32 and 34°C). Throughout this four year study, the level of hypothermia was strictly controlled, with a target temperature of 36°C, and active cooling was instituted if the temperature exceeded 37°C.

### Statistical analysis

We performed a retrospective observational study that included all resuscitated OHCAs from January 1, 2009 to December 31, 2012 in the Canton of Geneva, but excluded all resuscitated OHCA treated and transported by the helicopter emergency service.

The CSV file of the cardiac arrest data from the Geneva OHCA register (documented in accordance with the Utstein 2015 template [22]), was imported into the Stata 14.0 software (StataCorp, College Station, TX USA). All data was analysed using the same software.

Only information related to patient survival and destination following hospital discharge (domicile, nursing home etc.) was available, but not the patient’s precise neurological status.

Depending on the estimated theoretical sample sizes, Chi square or Fisher exact tests were used to compare proportions of categorical variables. The Student t-test (Welch correction) was used for continuous variables, where the distribution of the groups was normal; where the distribution of the groups was not normal, the non-parametric Mann-Whitney-Wilcoxon test was preferred. If a large inequality in the distribution of data was found between the groups, a logarithmic transformation of the continuous variables was performed before testing. When there were more than two groups compared in the tables, each group was always compared to the first group, which was considered as the reference group. A test was considered significant when p <0.05.

When analysing the differences between periods 1 and 2, and notably following implementation of the 2010 ACLS Guidelines, univariate logistic regressions were made between the variables that significantly influence survival rates after hospital discharge, but which were not modified *a priori* by the implementation of the 2010 ACLS Guidelines (patient characteristics, situation at the time the OHCA occurs, emergency medical dispatch, response times). During this analysis we undertook research into the collinearity of the aforementioned variables, based on current knowledge related to the different links between each of the variables. These same variables, except collinear variables, were included in a multivariable logistic regression model to adjust the effect of the differences between periods 1 and 2 on survival rates following hospital discharge. When the log linearity hypothesis related to regression coefficients of the continuous variables was not respected, these variables were categorized; then an overall p-value for all categories was reported. Finally, the “goodness of fit” to the model was checked globally using the Hosmer-Lemeshow test.

### Ethics

The creation of the Geneva OHCA register and the studies based on it, were approved by the Cantonal Commission for Research Ethics of Geneva. The Cantonal Commission for Research Ethics of Geneva waived the requirement for informed consent. The data were also analysed anonymously.

## Results

The 1537 OHCAs which occurred in the Canton of Geneva between 2009 and 2012 were registered in the OHCA register of Geneva. During the study period, there were only 869 OHCA for which resuscitation was attempted. The annual incidence of resuscitated OHCA was therefore measured at 46.7 / 100,000-inhabitants / year.

**Fig 2:**
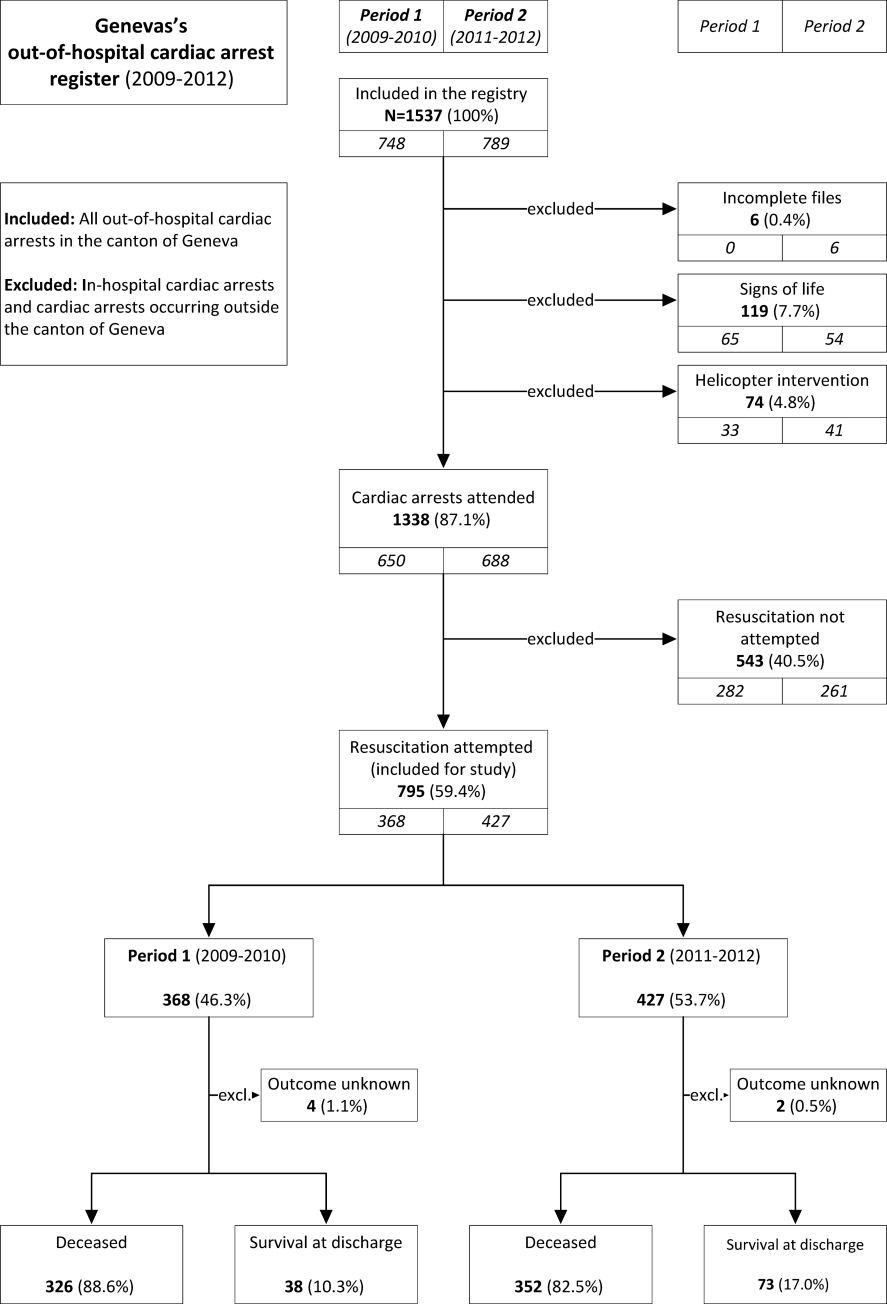
Registration flow chart and survival up to hospital discharge (2009–2012).

The very small number of patients transported by helicopter remained stable between the two periods studied (Fig 2), but helicopter emergency service forms were incomplete and were consequently excluded. Finally, 795 OHCAs were used in this study, i.e. 368 in period 1) and 427 in period 2) (Fig 2).

The detection of a cardiac arrest during the emergency phone call, the use of a laryngeal mask (introduced in 2010) and the use of an external automated cardiac massage device (introduced in 2011) did not appear to be associated with patient survival rates at the time of hospital discharge, or with the ROSC or the patient admission rate to hospital. Effectiveness of CPR provided by a non-professional observer and the time at which the first electric shock was administered were imprecisely documented. Dispatcher-assisted telephone CPR was not routinely proposed and was not documented. Therefore, these prognostic factors were not included in the analysis.

Table 1 lists the variables influencing survival rates at the time of hospital discharge and measures their evolution between periods 1 and 2. We found no statistically significant change between the two periods studied, either in the prognostic factors associated with patient typologies or in the situation at the time OHCA occurred. Only the use of CPR and automated external defibrillators (AED) by bystander witnesses increased significantly.

**Table 1:** Baseline characteristics of patients studied, features and interventions during cardiac arrest events.

Prognostic factors	Total			
		Period 1	Period 2	*p*
		*2009–2010*	*2011–2012*	
**Resuscitation attempted**	**795**	**368**	**427**	
**Age,** years (±SD)	64.5 ± 19.3	63.7 ± 19.0	65.2 ± 19.5	*0*.*296*
**Male sex**, n (%)	535 (67.3)	251 (68.2)	284 (66.5)	*0*.*611*
**On-going CPR at hospital admission**	37 (10.2)	11 (7.5)	26 (12.0)	*0*.*170*
**Detection of cardiac arrest by dispatchers**	731 (91.9)	223 (66.6)	274 (69.2)	*0*.*448*
**Pathogenesis (presumed aetiology)**, *n (%)*				
*Presumed traumatic aetiology*, *drowning and asphyxia*	70 (8.8)	32 (8.7)	38 (8.9)	
*Medical (not cardiac)*, *drug overdose*	95 (12.0)	47 (12.8)	48 (11.2)	*0*.*633*
*Presumed cardiac aetiology*	131 (16.5)	53 (14.4)	78 (18.3)	*0*.*472*
*Not documented aetiology*	499 (62.8)	236 (64.1)	263 (61.6)	*0*.*804*
**First monitored rhythm**, *n (%)*				
*Asystole*	410 (51.6)	195 (53.1)	215 (51.6)	
*PEA*	219 (27.6)	105 (28.6)	114 (26.7)	*0*.*927*
*VF/VT*	165 (20.8)	67 (18.3)	98 (23.0)	*0*.*130*
**Arrest location**, *n (%)*				
*Home*	468 (58.9)	218 (59.2)	250 (58.6)	
*Public location*	239 (30.1)	111 (30.2)	128 (30.0)	*0*.*972*
*Ambulance*	88 (11.1)	39 (10.6)	49 (11.5)	*0*.*696*
**Witnessed arrest**, *n (%)*				
*Un-witnessed arrest*,	209 (26.3)	99 (27.0)	110 (26.3)	
*Bystander witnessed*	497 (62.5)	229 (62.4)	268 (62.6)	*0*.*753*
*EMS witnessed*	88 (11.1)	39 (10.6)	49 (11.1)	*0*.*630*
**Bystander response** *(without EMS witnessed)*, *n (%)*	**707**	**329**	**378**	
*Bystander CPR performed*	223 (31.5)	**90 (27.4)**	**133 (35.2)**	***0*.*025***
*Bystander AED used*	25 (3.5)	**6 (1.8)**	**19 (5.0)**	***0*.*021***
**EMS response**	**795**	**368**	**427**	
*Response times*, *minutes / second (± SD)*	9’14” ± 4’08”	9’12” ± 4’13”	9’15” ± 4’03”	*0*.*548*
*Shock delivered*, *n (%)*	235 (29.6)	103 (28.0)	132 (30.9)	*0*.*368*
*Prehospital alarm system for suspected heart infarct performed*, *n (%)*	77 (9.7)	31 (8.4)	46 (10.8)	*0*.*264*
*Vascular access (intravenous or intraosseous) performed*, *n (%)*	728 (91.6)	336 (91.3)	392 (91.8)	*0*.*801*
*Endotracheal tube performed*, *n (%)*	645 (81.1)	304 (82.6)	341 (79.9)	*0*.*323*
*Adrenaline*, *number of times*, *n (%)*	658 (82.8)	313 (85.05)	345 (80.8)	*0*.*113*
*Adrenaline dosage*, *mg*	6.4 ± 4.5	**7.1 ± 4.9**	**5.8 ± 4**	***0*.*003***
*Atropine*, *number of times*, *n (%)*	136 (43.5)	**251 (68.2)**	**95 (22.3)**	***<0*.*001***
*Atropine dosage*, *mg*	2.3 ± 1.0	**2.6 ± 0.9**	**1.6 ± 1**	***<0*.*001***
*Amiodarone*, *number of times*, *n (%)*	219 (27.6)	99 (26.9)	120 (28.1)	*0*.*706*
*Amiodarone dosage*, *mg*	339 ± 121	366 ± 126	318 ± 113	***0*.*007***
*Aspirin*, *number of times*, *n (%)*	287 (36.1)	124 (33.7)	163 (38.2)	*0*.*190*
*Heparin*, *number of times*, *n (%)*	261 (32.8)	121 (32.9)	140 (32.8)	*0*.*978*
*Sodium Bicarbonate*, *number of times*, *n (%)*	112 (14.1)	**96 (26.1)**	**16 (3.8)**	***<0*.*001***
*Calcium Chloride*, *number of times*, *n (%)*	22 (2.8)	14 (3.8)	8 (1.9)	*0*.*098*
*Magnesium Sulphate*, *number of times*, *n (%)*	56 (7.0)	21 (5.7)	35 (8.2)	*0*.*098*
*Glucose*, *number of times*, *n (%)*	41 (5.2)	25 (6.8)	16 (3.8)	*0*.*053*

EMS: Emergency Medical System. PEA: Pulseless Electrical Activity. VF/ VT: Ventricular Fibrillation/ Ventricular Tachycardia. CPR: Cardiopulmonary Resuscitation. AED: Automated External Defibrillator. All variables given as numbers (group percentages in parenthesis) except age, response times, adrenaline dosage, atropine dosage and amiodarone. Figures are shown in column percentages.

Table 2 presents the univariate analysis of impact of the prognostic factors at the time of hospital discharge for the both periods as a whole (univariate results for secondary outcomes are reported in **S1 Table**).

**Table 2:** Influence of the prognostic factors at the time of hospital discharge (univariate analysis for both periods as a whole).

Prognostic Factors	Dead	Discharge alive	*p*
**Total, for resuscitations attempted (n = 795)**	**678 (85.9)**	**111 (14.1)**	
**Age,** years *(±SD)*	65.7 ± 19.6	56.4 ± 19.3	***<0*.*001***
**Male sex**, n (%)	442 (83.6)	87 (16.4)	***0*.*006***
**Pathogenesis (presumed aetiology)**, n (%)			
*Presumed traumatic aetiology*, *drowning and asphyxia*	64 (91.4)	6 (8.6)	
*Medical (not cardiac)*, *drug overdose*	79 (84.0)	15 (16.0)	*0*.*161*
*Presumed cardiac aetiology*	71 (54.2)	60 (45.8)	***<0*.*001***
*Not documented aetiology*	469 (94.0)	30 (6.0)	*0*.*403*
**First monitored rhythm**, n (%)			
*Asystole*	398 (97.3)	11 (2.7)	
*PEA*	188 (86.6)	29 (13.4)	***<0*.*001***
*VF/VT*	91 (56.2)	71 (43.8)	***<0*.*001***
**Arrest location**, n (%)			
*Home*	435 (93.0)	33 (7.1)	
*Public location*	181 (77.0)	54 (23.0)	***<0*.*001***
*Ambulance*	62 (72.1)	24 (27.9)	***<0*.*001***
**Witnessed arrest**, n (%)			
*Unwitnessed arrest*,	199 (95.7)	9 (4.3)	
*Bystander witnessed*	416 (84.2)	78 (15.8)	***<0*.*001***
*EMS witnessed*	62 (72.1)	24 (27.9)	***<0*.*001***
**Bystander response** *(without EMS witnessed)*, n (%)	**616 (87.6)**	87 (12.4)	
*Bystander CPR performed*	172 (77.8)	49 (22.2)	***<0*.*001***
*Bystander AED used*	14 (56.0)	11 (44.0)	***<0*.*001***
**EMS response**			
*Response times*, *minutes / seconds (±SD)*	9’27” ± 4’15”	7’45” ± 2’36”	***<0*.*001***
*Shock delivered*, n (%)	170 (73.6)	61 (26.4)	***<0*.*001***
*Pre-hospital alarm system for suspected heart infarct performed*, n (%)	33 (42.9)	44 (57.1)	***<0*.*001***
*Vascular access (intravenous or intraosseous) performed*, n (%)	613 (84.9)	109 (15.1)	***0*.*002***
*Endotracheal tube performed*, n (%)	556 (87.0)	83 (13.0)	*0*.*072*
*Adrenaline*, *number of times*, n (%)	586 (89.9)	66 (10.1)	***<0*.*001***
*Adrenaline dosage*, *mg*	6.7 ± 4.5	3.7 ± 3.8	***<0*.*001***
*Atropine*, *number of times*, n (%)	320 (93.6)	22 (6.4)	***<0*.*001***
*Atropine dosage*, *mg*	2.4 ± 1	1.8 ± 1.3	***0*.*011***
*Amiodarone*, *number of times*, n (%)	159 (74.0)	56 (26.0)	***<0*.*001***
*Amiodarone dosage*, *mg*	342 ± 118	328 ± 124	*0*.*389*
*Aspirin*, *number of times*, n (%)	213 (75.0)	71 (25.0)	***<0*.*001***
*Heparin*, *number of times*, n (%)	199 (76.8)	60 (23.2)	***<0*.*001***
*Sodium Bicarbonate*, *number of times*, n (%)	105 (94.6)	6 (5.4)	***0*.*005***
*Calcium Chloride*, *number of times*, n (%)	22 (100.0)	0 (0.0)	*0*.*034*
*Magnesium Sulphate*, *number of times*	41 (73.2)	15 (26.8)	***0*.*005***
*Glucose*, *number of times*, n (%)	40 (100.0)	0 (0.0)	***0*.*002***

EMS: Emergency Medical System. ROSC: Return Of Spontaneous Circulation. PEA: Pulseless Electrical Activity. VF/ VT: Ventricular Fibrillation/ Ventricular Tachycardia. CPR: Cardiopulmonary Resuscitation. AED: Automated External Defibrillator. For more than two groups, each group was always compared to the first group, which was considered as the reference group. Figures are shown in row percentages.

On the univariate analysis, the study showed that bystander witnesses only performed CPR on one third of the resuscitated OHCAs, but that their intervention doubled survival rates.

Vascular access, performed in more than 85% of resuscitated OHCAs, correlated favourably with the three outcomes measured. Adrenaline, was given to more than 80% of resuscitated OHCAs and Atropine was given to more than 40%. Amiodarone was administered to more than one quarter of the resuscitated OHCAs. It was noted that these drugs were administered less frequently and at lower doses for the group of survivors at the time of hospital discharge.

Orotracheal intubation was performed on more than 80% of the resuscitated OHCA patients which appeared to provide some initial benefit to ROSC and patients admitted to hospital, although ultimately it was not apparent that this influenced on patient survival rates at the time of hospital discharge.

Table 3 presents the univariate analysis, and show that the prognosis for OHCA patients at the time of hospital discharge clearly improved, with a survival rate in period 2 of 17.01% ([CI 95%: 13.8–21.07], n = 73) compared to the survival rate in period 1 which was 10.33% ([CI 95%: 7.68–14.03], n = 38). The number of OHCA patients admitted to hospital, compared to the number admitted in the previous period, also improved in 2011–2012 (p = 0.002). Moreover, there was no significant increase in ROSC. The number of patients transported to hospital with on-going cardiac massage increased by 7.5% ([95% CI: 3.25–11.80], n = 11) for period 1 to 12.0% ([95% CI: 7.66–16.30], n = 26) for period 2. This increase was not statistically significant (Table 1).

**Table 3:** Comparative outcomes for periods 1 and 2 (univariate analysis).

Prognostic factor groups	Any ROSC			Admitted			Discharge alive		
	Period 1	Period 2	*p*	Period 1	Period 2	*p*	Period 1	Period 2	*p*
**Resuscitation attempted**, n (%)	154 (41.9)	199 (46.7)	*0*.*178*	**146 (39.7)**	**217 (50.8)**	***0*.*002***	**38 (10.3)**	**73 (17.0)**	***0*.*007***
**Pathogenesis (presumed aetiology**), n (%)			* *						
*Presumed traumatic aetiology*, *drowning and asphyxia*	18 (11.7)	21 (10.6)	*0*.*934*	18 (12.3)	23 (10.6)	*0*.*717*	3 (7.9)	3 (4.1)	*0*.*577*
*Medical (not cardiac)*, *drug overdose*	24 (15.6)	32 (16.1)	*0*.*122*	**21 (14.4)**	**39 (18.0)**	***<0*.*001***	5 (13.2)	10 (13.7)	*0*.*159*
*Presumed cardiac aetiology*	45 (29.2)	68 (34.2)	*0*.*711*	44 (30.1)	69 (31.8)	*0*.*374*	**18 (45.4)**	**42 (57.5)**	***0*.*025***
*Not documented aetiology*	67 (43.5)	78 (39.2)	*0*.*755*	63 (43.2)	86 (39.6)	*0*.*143*	12 (31.6)	18 (24.7)	*0*.*43*
**First monitored rhythm**, n (%)			* *						
*Asystole*	60 (39.0)	58 (29.1)	*0*.*397*	52 (35.6)	64 (29.5)	*0*.*486*	4 (10.5)	7 (9.6)	0.327
*PEA*	**46 (29.9)**	**67 (33.7)**	***0*.*027***	**47 (32.2)**	**72 (33.2)**	***0*.*006***	9 (23.7)	20 (27.4)	0.057
*VF/VT*	48 (31.2)	74 (37.2)	*0*.*578*	47 (32.2)	81 (37.3)	*0*.*059*	25 (65.8)	46 (63.0)	*0*.*26*
**Arrest location**, n (%)			* *						
*Ambulance*	26 (16.9)	36 (18.1)	*0*.*487*	25 (17.1)	37 (17.1)	*0*.*244*	**6 (16.2)**	**18 (24.7)**	***0*.*036***

EMS: Emergency Medical System. PEA: Pulseless Electrical Activity. VF/ VT: Ventricular Fibrillation/ Ventricular Tachycardia. CPR: Cardiopulmonary Resuscitation. AED: Automated External Defibrillator. Figures are shown in column percentages.

Table 4 presents the results of the multivariable adjustment logistic regression model (the results of the univariate logistic regressions are shown in **S2 Table**). We excluded the variable "Arrest location" because of collinearity with the variables "Witnessed arrest" and "Response times".

**Table 4:** Multivariable logistic regression analysis of prognostic factors on survival rates at hospital discharge.

Prognostic factors	Adjusted OR		
	OR	IC 95%	*P*
**Period**			
*Period 1 (2009–2010)*	(1) Ref.		
*Period 2 (2011–2012)*	**1.87**	[1.08–3.22]	***0*.*025***
**Age group**			
*0–40 years*	(1) Ref.		
*40–55 years*	**0.98**	[0.39–2.46]	
*55–70 years*	**0.64**	[0.26–1.53]	
*70–85 years*	**0.24**	[0.09–0.60]	
*> 85 years*	**0.06**	[0.01–0.32]	***<0*.*001***
**Gender**			
*Female*	(1) Ref.		
*Male*	**0.97**	[0.53–1.77]	*0*.*911*
**Pathogenesis (presumed aetiology)**			
*Not documented aetiology*	(1) Ref.		
*Presumed traumatic aetiology*, *drowning and asphyxia*	**0.57**	[0.18–1.80]	*0*.*335*
*Presumed medical (not cardiac) aetiology and drug overdose*	**2.50**	[1.11–5.63]	***0*.*027***
*Presumed cardiac aetiology*	**5.00**	[2.57–9.74]	***<0*.*001***
**First monitored rhythm**			
*Asystole*	(1) Ref.		
*PEA*	**4.19**	[1.55–7.70]	***0*.*001***
*VF/VT*	**14.28**	[5.18–24.64]	***<0*.*001***
**Witnessed arrest**			
*Unwitnessed arrest*	(1) Ref.		
*Bystander or EMS witnessed*	**1.62**	[0.69–3.82]	*0*.*268*
**Bystander response**			
*Bystander CPR not performed*	(1) Ref.		
*Bystander CPR performed*	**1.39**	[0.78–2.45]	*0*.*260*
**Bystander AED use**			
*Automated external defibrillator (AED) not used*	(1) Ref.		
*Automated external defibrillator (AED) used*	**2.11**	[0.77–5.73]	*0*.*144*
**Response times**			
*0–2 min (EMS witnessed)*	(1) Ref.		
*2–6 min*	**1.38**	[0.56–3.40]	
*6–9 min*	**1.01**	[0.47–2.18]	
*9–12 min*	**0.53**	[0.21–1.34]	
*> 12 min*	**0.32**	[0.11–0.94]	***0*.*047***

EMS: Emergency Medical System. PEA: Pulseless Electrical Activity. VF/ VT: Ventricular Fibrillation/ Ventricular Tachycardia. CPR: Cardiopulmonary Resuscitation. AED: Automated External Defibrillator.

The univariate logistic regression odds ratio was measured at 1.78 [95% CI: 1.17–2.71] for survival at the time of hospital discharge for period 2) compared to period 1). The multivariable adjustment logistic regression model odds ratio was measured at 1.87 [CI95%: 1.08–3.22] for survival at the time of hospital discharge for period 2) compared to period 1). The difference between the coefficients was less than 15% and the model enhanced the effect of period 2) on survival. The logistic regression adjustment model covering the influence of the “before and after” period therefore confirmed that the difference in survival rates found in the univariate analysis between the periods studied persisted following adjustment for all the confounding factors identified. The Hossmer-Lemeshow test (p = 0.381) validates the model, which match the expected events and the events observed in the data analysed.

## Discussion

The prognosis for survival of OHCA patients at the time of hospital discharge between 2009 and 2012 improved considerably in the two years following the implementation of the 2010 ACLS Guidelines, thus achieving one of the best survival rates (17.01%) published for this period. We were able to show that this improvement in survival rate was not affected by the variables included in the adjustment model (patient characteristics, situation at the time the OHCA occurs, emergency medical dispatch, and response times).

We also examined any changes in treatment provided and special care administered by EMS personnel (these were not included in the adjustment model) in order to assess what the impact these changes might have on survival rates. Following the implementation of the 2010 ACLS Guidelines, it Geneva EMS personnel administered less Adrenaline and Amiodarone than previously, it being noted that their effect on survival is highly debated. Furthermore, Geneva EMS personnel no longer administer sodium bicarbonate or atropine, which it is now agreed have no impact on survival rates. As has been found by other authors [24, 25] we found no specific association with survival rates at hospital discharge following the introduction in 2011 of the external automated cardiac massage device. The measures provided by Geneva EMS personnel that do appear to have any real correlation with the outcome of an OHCA (triggering a pre-hospital alarm, establishing vascular access, and delivering an electric shock) did not change between the two periods examined. In conclusion, the aforementioned changes do not appear to play a significant role on survival rates.

There is no immediately apparent reason that explains the recorded level of improvement in survival rates following implementation of the 2010 ACLS Guidelines. What did appear to be relevant however was the increased number of patients transported to hospital and who were still alive at hospital discharge and not the rate of ROSC before transportation. We suggest therefore, that it is the care provided during the CPR phase or after returning to spontaneous circulation which is responsible for the improvement in survival rates.

We were unfortunately not in a position to measure the quality of chest compressions provided by EMS staff or bystanders, nor the interruptions of chest compressions during CPR. Only a monitoring of the quality of chest compressions provided by EMS staff could have shown us that it was this care that had an impact on the survival rate at hospital discharge. Indeed, "the importance of early and uninterrupted chest compressions" was one of the highlights of the 2010 guideline revisions [12]. Although we understand that they were not able to measure the quality of CPR, the Japanese study also found improved survival rates following implementation of the 2010 Guidelines for witnessed OHCA, and the authors suggest that the emphasis on "high-quality CPR" may have improved survival rates [26]. Despite the fact that we were unable to measure the quality of CPR, we would be tempted to suggest the same. In addition, Geneva Emergency Medical Services personnel have likely applied the additional phase of "immediate post-resuscitation care" to the "survival chain" with the development of "high-quality resuscitation" [17, 27]. Refresher training in CPR and resuscitation introduced following implementation of the 2010 Guidelines, for all EMS personnel, may well have had an effect on EMS personnel’s attention to care. The improvement observed is perhaps a "surfing on the guidelines’ wave" [28]. These two elements may well explain the improved survival rates observed in our ALS system.

Several studies have examined the impact of implementing the 2005 Guidelines [6, 19, 20] and found that the overall prognosis for survival was slightly improved [29], reaching 9% [16, 30] to 13% [19] in 2009–2010. A survival rate of 10.33% measured in Geneva during period 1, is similar to that published in other studies related to EMS worldwide where ALS systems are implemented [5, 31–33]. From 2009 to 2012, the survival rate improved for all OHCA patients [33, 34], with survival rates of presumed cardiac aetiology oscillating between 12% and 14% throughout 2012 [32]. Two studies reported similar survival rates which correspond to those found in period 2 of this study: an American report, covering the period 2012–2013, but which had a very different proportion of shockable rhythms (> 30%) which may explain the relatively high survival rates [35]. The other was a German report, which covered the period between 2011 and 2014, but which only describes non-traumatic OHCAs [36].

The authors believe that this is the first study that correlates the evolution of survival at the time of hospital discharge with the implementation of the 2010 ACLS Guidelines in a European EMS system, which only provides an ALS response. In particular, the number of ROSC and patients admitted to hospital increased during period 2, and were proportionately also significantly higher than in the other studies [32, 36], whereas the essential baseline of patients characteristics and the context at the time OHCA occurred had not changed. In a similar, but considerably larger population-based study from Japan, the impact of the implementation of the 2010 Guidelines was examined [26]. However, the aforementioned study was based on an “intermediate life support” system, not an ALS system. In this study, the Japanese researchers found that survival rates at one-month following hospital discharge improved after implementation of these guidelines. It would appear that, in the same population, the improvement is also greater when OHCA is observed by EMS personnel [37]. In the Japanese EMS system, where only life-saving technicians are present, treatment provided is limited. This is a somewhat different system to Geneva, where both paramedics and physicians provide ACLS immediately.

### Limitations

There are several limitations to our study, due to the fact that it was a short-term, observational, retrospective, mono-centric study with a limited number of cases. No documentation related to Dispatcher assisted telephone CPR was available. No monitoring equipment was available to directly measure the quality of chest compressions. There was little change in intra-hospital care between 2009 and 2012 and this was confirmed by Intensive Care Unit staff (including treatment cessation decision criteria) although we do no hold more precise information. There was however an increase in the number of patients admitted to hospital, but recognise that the lack of precise information concerning post-resuscitation intra hospital management remains a limiting factor.

**Of note**: Table 4 shows that, patient prognosis is improved when the response time is 2–6 min rather than 0–2 min. In all cases where the response time was 0–2 min, the OHCAs had taken place in the presence of EMS personnel. Whilst we are not able to explain this difference in outcome, we suggest that the aetiologies of these OHCA occurring in front of "EMS witnesses" were different from those aetiologies of other OHCAs, and that the survival associated with these different aetiologies depended less on the response time.

An unmeasured confounding factor that might modify the results remains possible. Finally, the lack of available data on the neurological status of patients at the time of hospital discharge limits the scope of our results.

## Conclusions

This study demonstrated that survival rate after out-of-hospital cardiac arrest improved significantly in the period following the implementation of the 2010 Guidelines. The adjustment model suggests that this may be associated with the improvement in the quality of care. The actual quality of care provided by EMS personal, through the use of tools for quality measurement of chest compressions and other quality measurement tools, should be evaluated in subsequent studies.

## Supporting information

S1 Table Univariate analysis for secondary outcomes. (PDF)

S2 Table Univariate logistic regression of prognostic factors on survival at hospital discharge. (PDF)

S1 Dataset Minimal dataset of the study. (XLS)
