# Supplementary material for: Improvement of out-of-hospital cardiac arrest survival rate after implementation of the 2010 resuscitation guidelines
Source: PLoS One. 2018 Sep 24;13(9):e0204169. doi: 10.1371/journal.pone.0204169 (PMC6152955; doi:10.1371/journal.pone.0204169)
Supplement: S1 Table — (PDF) [file pone.0204169.s001.pdf]

S1 Table. Univariate analysis for secondary outcomes

Prognostic Factors	ROSC			Transported to hospital		
	Without ROSC	Any ROSC	*p*	Not admitted	Admitted	*p*
**Total, for resuscitation attempted (n=795)**	**442 (55.6)**	**353 (44.4)**		**432 (54.3)**	**363 (45.7)**	
**Age,** years *(±SD)*	65.5 ± 19.8	63.3 ± 18.6	*0.117*	67.1 ± 18.3	61.4 ± 20.0	***<0.001***
**Male sex**, n (%)	281 (52.5)	254 (47.5)	***0.012***	274 (51.2)	261 (48.8)	***0.011***
**Pathogenesis (presumed aetiology)**, n (%)						
Presumed traumatic aetiology, drowning and asphyxia	31 (44.3)	39 (55.7)		29 (41.4)	41 (58.6)	
Medical (not cardiac), drug overdose	39 (41.1)	56 (58.9)	*0.678*	35 (36.8)	60 (63.2)	***0.055***
Presumed cardiac aetiology	18 (13.7)	113 (86.3)	***<0.001***	18 (13.7)	113 (86.3)	***<0.001***
Not documented aetiology	354 (70.9)	145 (29.1)	***<0.002***	350 (70.1)	149 (29.9)	***<0.001***
**First monitored rhythm**, n (%)						
Asystole	292 (71.2)	118 (28.8)		294 (71.7)	116 (28.3)	
PEA	106 (48.4)	113 (51.6)	***<0.001***	100 (45.7)	119 (54.3)	***<0.001***
VF/VT	43 (26.1)	122 (73.9)	***<0.001***	37 (22.4)	128 (77.6)	***<0.001***
**Arrest location**, n (%)						
Home	312 (66.7)	156 (33.3)		311 (66.5)	157 (33.5)	
Public location	104 (43.5)	135 (56.5)	***<0.001***	95 (39.7)	144 (60.3)	***<0.001***
Ambulance	26 (29.6)	62 (70.4)	***<0.001***	26 (29.5)	62 (70.5)	***<0.001***
**Witnessed arrest**, n (%)						
Unwitnessed arrest,	167 (79.9)	42 (20.1)		162 (77.5)	47 (22.5)	
Bystander witnessed	248 (49.9)	249 (50.1)	***<0.001***	243 (48.9)	254 (70.0)	***<0.001***
EMS witnessed	26 (29.6)	62 (70.4)	***<0.001***	26 (29.5)	62 (70.5)	***<0.001***
**Bystander response** *(without EMS witnessed)*, n (%)	**416 (58.8)**	291 (41.2)		**406 (57.4)**	301 (42.6)	
Bystander CPR performed	101 (45.3)	122 (54.7)	***<0.001***	101 (45.3)	122 (54.7)	***<0.001***

Bystander AED used	8 (32.0)	17 (68.0)	***0.005***	7 (28.0)	18 (72.0)	***0.002***
**EMS response**						
Response times, minutes / seconds (±SD)	9’14’’ ± 3’37’’	9’14’ ± 4’46’’	*0.391*	9’21’’ ± 3’46’’	9’04’’ ± 4’33’’	*0.104*
Shock delivered, n (%)	91 (38.7)	144 (61.3)	***<0.001***	84 (35.7)	151 (64.3)	***<0.001***
Prehospital alarm system for suspected heart infarct performed, n (%)	6 (7.8)	71 (92.2)	***<0.001***	3 (3.9)	74 (96.1)	***<0.001***
Vascular access (intravenous or intraosseous) performed, n (%)	384 (52.7)	344 (47.3)	***<0.001***	373 (51.2)	355 (48.8)	***<0.001***
Endotracheal tube performed, n (%)	340 (52.7)	305 (47.3)	***0.001***	330 (51.2)	315 (48.8)	***<0.001***
Adrenaline, number of times, n (%)	362 (55.0)	296 (45.0)	*0.469*	356 (54.1)	302 (45.9)	*0.769*
Adrenaline dosage, mg	6.6 ± 4.1	6.0 ± 5.0	***0.006***	6.8 ± 4.0	6.0 ± 5.0	***<0.001***
Atropine, number of times, n (%)	190 (54.9)	156 (45.1)	*0.733*	197 (56.9)	149 (43.1)	*0.197*
Atropine dosage, mg	2.4 ± 1	2.2 ± 1	*0.071*	2.5 ± 0.9	2.1 ± 1	***0.004***
Amiodarone, number of times, n (%)	78 (35.6)	141 (64.4)	***<0.001***	75 (34.3)	144 (65.8)	***<0.001***
Amiodarone dosage, mg	361 ± 109	327 ± 126	***0.04***	370 ± 111	323 ± 123	***0.007***
Aspirin®, number of times, n (%)	93 (32.4)	194 (67.6)	***<0.001***	99 (34.5)	188 (65.5)	***<0.001***
Heparin®, number of times, n (%)	93 (35.6)	168 (64.4)	***<0.001***	97 (37.2)	164 (62.8)	***<0.001***
Sodium Bicarbonate, number of times, n (%)	66 (58.9)	46 (41.1)	*0.444*	68 (60.7)	44 (39.3)	*0.144*
Calcium Chloride, number of times, n (%)	15 (68.2)	7 (31.8)	*0.162*	16 (72.7)	6 (27.3)	***0.06***
Magnesium Sulphate, number of times	20 (35.7)	36 (64.3)	***0.002***	20 (35.7)	36 (64.3)	***0.004***
Glucose, number of times, n (%)	29 (70.7)	12 (29.3)	***0.045***	29 (70.7)	12 (29.3)	***0.03***

EMS: Emergency Medical System. ROSC: Return Of Spontaneous Circulation. PEA: Pulseless Electrical Activity. VF/ VT: Ventricular Fibrillation/ Ventricular Tachycardia.

CPR: Cardiopulmonary Resuscitation. AED: Automated External Defibrillator.

Figures are shown in row percentages.
