# Supplementary material for: Improvement of out-of-hospital cardiac arrest survival rate after implementation of the 2010 resuscitation guidelines
Source: PLoS One. 2018 Sep 24;13(9):e0204169. doi: 10.1371/journal.pone.0204169 (PMC6152955; doi:10.1371/journal.pone.0204169)
Supplement: S2 Table — (PDF) [file pone.0204169.s002.pdf]

S2 Table. Univariate logistic regression analysis of prognostic factors on survival at hospital discharge

Prognostic factors	Not adjusted OR		
	OR	IC 95%	*P*
**Period**			
Period 1 (2009-2010)	(1) Ref.		
Period 2 (2011-2012)	**1.78**	[1.17- 2.71]	***0.007***
**Age group**			
0-40 years	(1) Ref.		
40-55 years	**1.40**	[0.71- 2.75]	
55-70 years	**1.05**	[0.55- 1.99]	
70-85 years	**0.41**	[0.21- 0.83]	
> 85 years	**0.10**	[0.02- 0.47]	***<0.001***
**Gender**			
Female	(1) Ref.		
Male	**1.94**	[0.18- 1.14]	***0.007***
**Pathogenesis (presumed aetiology)**			
Not documented aetiology	(1) Ref.		
Presumed traumatic aetiology, drowning and asphyxia	**1.45**	[0.58- 3.62]	
Presumed medical (not cardiac) aetiology and drug overdose	**2.94**	[1.51- 5.71]	
Presumed cardiac aetiology	**13.07**	[7.89- 21.65]	***<0.001***
**First monitored rhythm**			
Asystole	(1) Ref.		
PEA	**5.58**	[2.73- 11.41]	***<0.001***
VF/VT	**28.23**	[14.38- 55.43]	***<0.001***
**Arrest location**			
Home	(1) Ref.		
Public location	**3.93**	[2.47- 6.27]	***<0.001***
Ambulance	**5.10**	[2.83- 9.20]	***<0.001***
**Witnessed arrest**			
Unwitnessed arrest	(1) Ref.		
Bystander or EMS witnessed	**4.72**	[2.34- 9.51]	***<0.001***
**Bystander response**			
Bystander CPR not performed	(1) Ref.		
Bystander CPR performed	**2.74**	[1.82-4.13]	***<0.001***
**Bystander AED use**			
Automated external defibrillator (AED) not used	(1) Ref.		
Automated external defibrillator (AED) used	**5.73**	[2.79- 11.75]	***<0.001***
**Response times**			
0-2 min	(1) Ref.		
2-6 min	**0.80**	[0.42-1.52]	
6-9 min	**0.56**	[0.33-0.96]	
9-12 min	**0.31**	[0.16-0.61]	
> 12 min	**0.23**	[0.10-0.54]	***<0.001***

EMS: Emergency Medical System. PEA: Pulseless Electrical Activity.
VF/ VT: Ventricular Fibrillation/ Ventricular Tachycardia.
CPR: Cardiopulmonary Resuscitation. AED: Automated External Defibrillator.
